# Complete mitochondrial genome of *Quercus variabilis* (Fagales, Fagaceae)

**DOI:** 10.1080/23802359.2019.1687027

**Published:** 2019-11-12

**Authors:** Quanxin Bi, Dongxing Li, Yang Zhao, Mengke Wang, Yingchao Li, Xiaojuan Liu, Libing Wang, Haiyan Yu

**Affiliations:** State Key Laboratory of Tree Genetics and Breeding, Key Laboratory of Tree Breeding and Cultivation, National Forestry and Grassland Administration, Research Institute of Forestry, Chinese Academy of Forestry, Beijing, China

**Keywords:** *Quercus variabilis*, mitochondrial genome, phylogenetic analysis

## Abstract

*Quercus variabilis* (Chinese cork oak) is an economically valuable oak as the source of industrial cork, which was widely distributed in eastern Asia. In this study, the complete mitochondrial genome of *Q. variabilis* was sequenced using the Illumina Hiseq and PacBio Sequel technique. The mitogenome is 412,886 bp in length and the GC content is 45.76%. The genome consists of 36 protein-coding genes, 3 ribosomal-RNA genes, and 21 transfer-RNA genes. Phylogenetic analysis based on protein-coding genes showed that *Q. variabilis* was close to the species in the Cucurbitaceae family.

*Quercus variabilis* (Chinese cork oak), the oak species, which is an economically valuable oak and one of the most important afforestation tree species in China is used as the source of industrial cork (Wang et al. 2016; Liu et al. 2018). In this study, we reported the complete mitogenome of *Q. variabilis* using high-throughput sequencing and PacBio sequencing technique. The annotated mitogenome of *Q. variabilis* has been deposited in GenBank under the accession number MN199236.

The sample of *Q. variabilis* adopted in this study was collected from Jiaozuo boai county of Henan Province, China (35°20′38.29″N, 113°09′42.98″E). The fresh leaves were collected and then frozen in liquid nitrogen and stored at −80 °C. The voucher specimens were deposited in the State Key Laboratory of Tree Genetics and Breeding, Research Institute of Forestry, Chinese Academy of Forestry, Beijing, China (voucher number: QV201905003). The mtDNA were isolated with an improved extraction method (Chen et al. 2011), and the mitochondrial genome were sequenced by Pacbio Sequel system and Illumina Hiseq system. Then, the genome were assembled by both the Pacbio Sequel data and the Illumina Hiseq data using SPAdes v3.10.1 (Antipov et al. 2016). The mitochondrial genes were annotated by homology alignments and *de novo* prediction.

The complete mitogenome of *Q. variabilis* is 412,886 bp in length, with the overall GC contents approximate to 45.76%. The mitogenome of *Q. variabilis* comprises of 36 protein-coding genes, 3 ribosomal-RNA genes, and 21 transfer-RNA genes. The total sequence length of protein-coding gene was 33,270 bp and the average length of genes was 924 bp.

The phylogenetic relationships of *Q. variabilis* and five other plant mitogenome were reconstructed using Bayesian analysis based on protein-coding genes (Figure 1). Sequences were aligned using MEGA X software (Kumar et al. 2018). Phylogenetic analysis confirmed that *Q. variabilis* was close to the species in the Cueurbitaceae family. All members in the phylogenetic tree provided the relationships among the Brassicales, Cucurbitales, Fagales, and Vitales.

**Figure 1.**
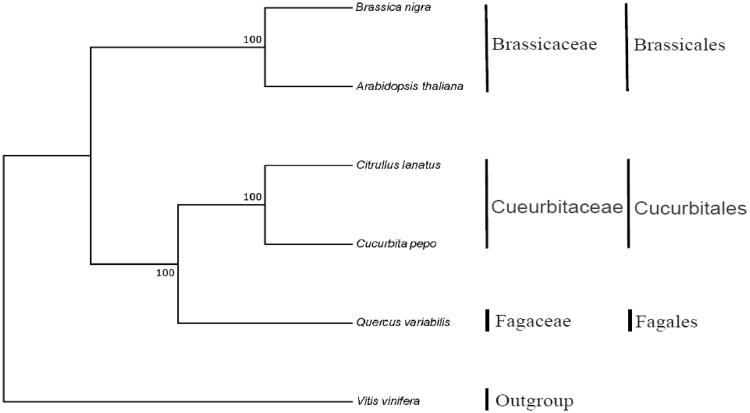
The phylogenetic tree based on six complete mitochondrial genomes. Accession number: *Arabidopsis thaliana* (Y08501), *Brassica nigra* (NC_029182), *Citrullus lanatus* (GQ856147), *Cucurbita pepo* (GQ856148), *Vitis vinifera* (NC012119.1), and *Quercus variabilis* (MN199236).
